# Triple Genetic Diagnosis in a Patient with Late-Onset Leukodystrophy and Mild Intellectual Disability

**DOI:** 10.3390/ijms25010495

**Published:** 2023-12-29

**Authors:** Domizia Pasquetti, Annalisa Gazzellone, Salvatore Rossi, Daniela Orteschi, Federica Francesca L’Erario, Paola Concolino, Angelo Minucci, Carlo Dionisi-Vici, Maurizio Genuardi, Gabriella Silvestri, Pietro Chiurazzi

**Affiliations:** 1 Section of Genomic Medicine, Department of Life Sciences and Public Health, Università Cattolica del Sacro Cuore, 00168 Rome, Italy; 2 Medical Genetics Unit, Fondazione Policlinico Universitario “A. Gemelli” IRCCS, 00168 Rome, Italy; 3 Department of Neurosciences, Faculty of Medicine and Surgery, Università Cattolica del Sacro Cuore, 00168 Rome, Italy; 4 Neurology Unit, Fondazione Policlinico Universitario “A. Gemelli” IRCCS, 00168 Rome, Italy; 5 Departmental Unit of Molecular and Genomic Diagnostics, Fondazione Policlinico Universitario “A. Gemelli” IRCCS, 00168 Rome, Italy; 6 Division of Metabolic Diseases, Bambino Gesù Children’s Hospital IRCCS, 00165 Rome, Italy

**Keywords:** polycystic kidney disease, phenylketonuria, leukodystrophy, CES, CMA, intellectual disability, 15q11.2 duplication

## Abstract

We describe the complex case of a 44-year-old man with polycystic kidney disease, mild cognitive impairment, and tremors in the upper limbs. Brain MRI showed lesions compatible with leukodystrophy. The diagnostic process, which included clinical exome sequencing (CES) and chromosomal microarray analysis (CMA), revealed a triple diagnosis: autosomal dominant polycystic kidney disease (ADPKD) due to a pathogenic variant, c.2152C>T-p.(Gln718Ter), in the *PKD1* gene; late-onset phenylketonuria due to the presence of two missense variants, c.842C>T-p.(Pro281Leu) and c.143T>C-p.(Leu48Ser) in the *PAH* gene; and a 915 Kb duplication on chromosome 15. Few patients with multiple concurrent genetic diagnoses are reported in the literature; in this ADPKD patient, genome-wide analysis allowed for the diagnosis of adult-onset phenylketonuria (which would have otherwise gone unnoticed) and a 15q11.2 duplication responsible for cognitive and behavioral impairment with incomplete penetrance. This case underlines the importance of clinical genetics for interpreting complex results obtained by genome-wide techniques, and for diagnosing concurrent late-onset monogenic conditions.

## 1. Introduction

Genetic clinicians nowadays perform genome-wide analysis on a regular basis, and must deal with a large number of genetic variants, both single-nucleotide variants (SNVs) and copy number variants (CNVs). The use of genome-wide technologies in the clinical setting will exponentially increase the number of patients diagnosed with multiple molecular alterations, some of which might have been suspected from the phenotype, while others may be incidental or confounding findings. These considerations are even more valid in the context of late-onset conditions; in fact, genetic diseases are frequently addressed in the differential diagnosis of adult patients in whom most disorders have a multifactorial origin [1]. Leukodystrophies are a group of inherited white matter disorders with a heterogeneous genetic background, phenotypic variability, and disease onset at variable age. They usually present in infants with non-specific features, such as intellectual disability and motor or coordination dysfunction, while adult-onset cases are more rare [2]. In adults, a broad range of conditions can result in the typical imaging patterns, including inflammatory, infectious, and malignant conditions, with significant involvement of small blood vessels. Once treatable and acquired causes of white matter disease are ruled out, usually patients are referred for genetic investigation. To date, more than 20 disease-causing genes are known, leading to autosomal, recessive and X-linked forms of inheritable leukodystrophies. Among these, the frequent overlap of clinical and radiological features can present a challenge in establishing a definitive diagnosis [3]. Here, we report the clinical and molecular findings of a young adult presenting with a subacute neurological condition and polycystic kidney disease (PKD); clinical exome sequencing (CES) and chromosomal microarray analysis (CMA) revealed three different molecular alterations potentially correlated with the clinical picture.

## 2. Results

**Figure 1:**
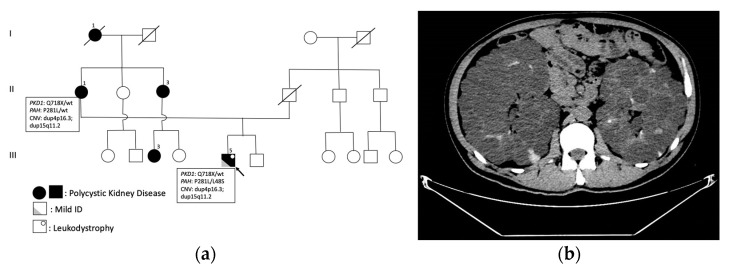
(**a**) Family tree showing autosomal dominant segregation of polycystic kidney disease: I-1, II-1, II-3, III-3 and III-5 are affected by PKD; the proband (III-5) is highlighted with a black arrow. (**b**) Computed tomography of the lower and upper abdomen showing enlarged kidneys with multiple hyperdense cystic formations.

**Figure 2:**
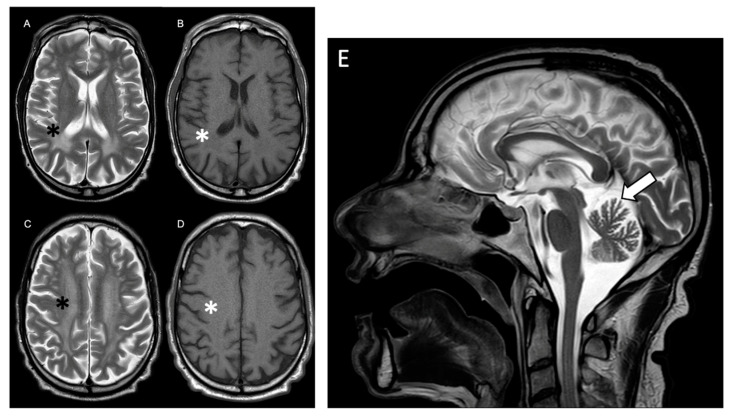
Panels showing T2-weighted (**A**,**C**) and T1-weighted (**B**,**D**) axial brain MRI scans at the basal ganglia (**A**,**B**) and corona radiata (**C**,**D**) level. An extensive and symmetric T2 hyperintensity (**A**,**C**) black asterisks involving mainly occipital and parietal white matter was observed, with corresponding T1 slightly hypointense signal (**B**,**D**) white asterisks, sparing subcortical U-fibers and corpus callosum. T2-weighted sagittal brain MRI scans (**E**) showing mild vermian cerebellar atrophy (white arrow).

Our patient is a 44-year-old male with a history of chronic renal failure secondary to polycystic kidney disease (Figure 1) and mild psychomotor delay. He is the first of two brothers born to non-consanguineous parents with a maternal family history of PKD, which had not been previously investigated. His father had died at the age of 62 because of colorectal cancer. Motor milestones were reached normally; but he experienced language delay and had poor school performance. His mother showed clinical signs of polycystic kidney disease at the age of 51, and underwent haemodialysis for 11 years until she had a kidney transplant at the age of 62. The patient’s neurological symptoms appeared at 42 years of age, with acute onset of spastic hypertonus and limb tremors. He recently experienced urinary incontinence and erectile dysfunction. Brain MRI showed a pattern compatible with leukodystrophy (Figure 2); two years later, he was hospitalized in our Neuropsychiatric Department because of his worsening clinical picture. Neurological examination showed spastic paraparesis with moderate lower limb weakness but marked spasticity (bilateral modified Ashwort scale = 3). No atrophy or fasciculations were noticed. Deep tendon reflexes were brisk (3+) at both upper and lower limbs, while Hoffman and Babinski signs were present bilaterally. Tone and strength were normal at upper limbs, and cranial nerves were intact. Cognitive tests confirmed mild intellectual disability. The neuropsychological assessment revealed below-average performance in all tests of verbal episodic memory (the Rey Auditory Verbal Learning Test) and visual episodic memory (the Rey–Osterrieth complex figure), in a spatial working memory task, in a graphic planning task, and in all language tests.

Spinal cord MRI was unremarkable, while brain MRI (Figure 2) showed extensive bilateral fluid-attenuated inversion recovery (FLAIR) hyperintensity involving subcortical white matter of the centrum semiovale and corona radiata, bilateral periventricular and peritrigonal region, and bilateral precentral and postcentral gyri. A corresponding T1 slightly hypointense signal in these regions, particularly in occipital white matter, was evident. The subcortical U-fibers and corpus callosum were spared. Also, mild cerebellar atrophy was detected. Diffusion-weighted imaging (DWI) and susceptibility-weighted imaging (SWI) scans were normal. MR spectroscopy did not show elevated levels of lactate. Gadolinium contrast was not infused because of PKD. Nerve conduction studies documented axonal sensory and demyelinating motor polyneuropathy, and EEG showed generalized background slowing in the theta frequency range; the fundus oculi was normal. Serology testing for HIV, syphilis, hepatitis B/C, tuberculosis, and JC virus was negative; the autoimmune antibody screening, blood lactic acid, folate and vitamin B12, and CSF examination were also normal. We therefore performed additional tests to exclude common causes of leukodystrophy (VLCFA levels, galactocerebrosidase and glycogen-branching enzyme activity, serum cholestanol, gonadotropins, free testosterone, homocysteine) were all normal [4]. At the time of our first genetic evaluation, the patient had already performed a multigene panel screening for leukodystrophies with normal results. Abdominal CT scan revealed enlarged kidneys with multiple hyperdense cystic formations (Figure 1B); moreover, multiple cysts were present in the liver. 

**Figure 3:**
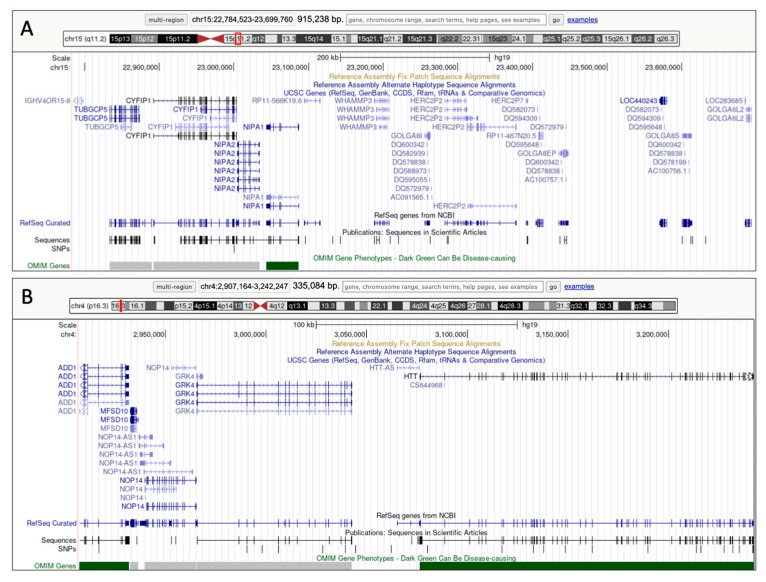
Panels showing chromosome 15q11.2 (**A**) and chromosome 4p16.3 (**B**) duplications, as reported in the UCSC Genome Browser (https://genome.ucsc.edu/, accessed on 20 November 2023) using assembly ID: GRCh37/hg19. The red frames indicate the localization of the duplicated regions on chromosome 15 and 4.

CES analysis eventually identified a pathogenic *PKD1* variant, c.2152C>T-p.(Gln718Ter), maternally inherited, and two pathogenic *PAH* variants: maternally inherited c.842C>T-p.(Pro281Leu), and c.143T>C-p.(Leu48Ser), likely paternal or de novo. The maternal variant is reported by ClinVar as likely pathogenic (https://www.ncbi.nlm.nih.gov/clinvar/variation/589, accessed on 10 November 2023), while the second variant is listed by ClinVar as pathogenic (https://www.ncbi.nlm.nih.gov/clinvar/variation/608, accessed on 10 November 2023). After identifying the *PAH* variants, phenylalanine levels in the patient’s blood were assessed, and were found to be highly increased (1592 μMol). Lastly, CMA analysis revealed a small 335 Kb duplication in 4p16.3 and a larger 915 Kb duplication in 15q11.2 (Figure 3).

## 3. Discussion

When a monogenic condition is suspected and the clinical phenotype is not suggestive of a specific causative gene, genome-wide strategies such as CMA (to look for CNVs) and CES or whole-exome sequencing (WES) are now commonly adopted to identify pathogenic variants. In this case, we performed both CMA and CES and found at least three different molecular alterations relevant to our patient’s phenotype. Until now, few patients with multiple genetic diagnoses have been reported [5], but co-occurrence of multiple monogenic conditions in complex patients will become more and more frequent.

In this young man, we first identified the *PKD1* variant responsible for the autosomal dominant polycystic kidney disease segregating in the maternal side of the family. Indeed, the well-known pathogenic c.2152C>T-p. (Gln718Ter) *PKD1* variant was also confirmed in the proband’s mother.

Furthermore, CES revealed the presence of two missense variants in the *PAH* gene, c.842C>T and c.143T>C, likely in trans since only the first variant was found in the mother; the second variant was either transmitted from the (deceased) father or arose de novo. This finding strongly suggested the diagnosis of phenylketonuria (PKU). PKU is an autosomal recessive metabolic condition due to biallelic pathogenic variants in the *PAH* gene, encoding phenylalanine hydroxylase [6]. PKU is mainly a childhood disorder, but in rare cases, the first symptoms occur in adulthood, as in this case, mimicking other neurological conditions [7,8]. A few patients with adult-onset untreated PKU are reported in the literature [9]; most of these patients, including ours, show a MRI cerebral leukodystrophy pattern. The importance of considering PKU in case of an adult-onset leukodystrophy stems from the availability of an effective treatment, even in late-diagnosed cases.

Both c.842C>T-p.(Pro281Leu) and c.143T>C-p.(Leu48Ser) are considered pathogenic *PAH* variants. The Pro281Leu, besides being a missense variant, is also supposed to alter splicing [10,11], whereas the Leu48Ser variant affects a highly conserved residue and is expected to disrupt PAH protein function [12]. These variants have been also reported in combination by the BioPku database [13] in 38 patients with either the mild (44.74%) or classic (55.26%) PKU phenotype. 

The age of onset and severity of PKU mostly depends on residual enzymatic activity, although genotype–phenotype correlation is not straightforward [14]. Some missense variants do not completely abolish enzymatic activity, leading to a nonclassical, milder PKU phenotype [15]. While the c.842C>T variant may completely abolish PAH activity because of its likely interference with splicing, the c.143T>C variant (supposedly inherited from the father) apparently has 39% of residual activity [16]. This may explain why diagnosing PKU in our patient has been so challenging, allowing the late onset of PKU after the age of 40. The diagnosis of PKU was biochemically confirmed by measuring blood levels of phenylalanine (20 times higher than normal in our patient).

However, our patient’s clinical history was characterized by early-onset mild intellectual disability and delayed language development, not consistent with the relatively spared enzymatic activity of phenylalanine hydroxylase. In order to evaluate other possible genetic factors responsible for this discrepancy, we performed CMA and uncovered two microduplications involving the 15q11.2 and 4p16.3 chromosomal regions (Figure 3). Actually, both CNVs had been also detected by CES, since both regions contain disease-causing genes, as analyzed by the SOPHiA Genetics Clinical Exome Solution^®^ v2 kit.

The 15q11.2 duplication (grch37 chr15: 22784523-23699760), located upstream of the PWS/AS region, harbors four highly conserved non-imprinted genes (*TUBGCP5*, *CYFIP1*, *NIPA1*, *NIPA2*) and has already been associated with cognitive, language, and behavioral impairment [17]. It is worth noting that *CYFIP1* encodes a cytoplasmic interactor of FMRP [18,19], the fragile X syndrome protein, and is involved in different neuronal processes such as actin filament reorganization [20] and intracellular signaling [21]. Duplications of 15q11.2 are inherited from an unaffected parent in approximately 80% of cases [22]; therefore, it was not surprising that the dup15q11.2 was also found in our patient’s mother. The role of dup4p16.3 is still unclear. It spans 335 Kb (grch37 chr4:2907164-3242247) and involves two disease-causing genes, *ADD1* and *HTT*, which are not related to the observed phenotype. Therefore, given its smaller size (335 vs. 915 Kb) and the limited gene content, it appears that the 15p11.2 duplication explains most of our patient’s mild intellectual disability.

In conclusion, this case demonstrates the relevant role of clinical genetics in analyzing the ever-growing amount of genomic data produced by NGS technology and correlating it with a complex phenotype comprising multiple genetic conditions. The adult onset of PKU in our patient further complicated the diagnosis, since one of the two missense *PAH* variants had a relatively large residual enzymatic activity. While NGS technology allowed the rapid and cost-effective identification of the molecular cause of ADPKD and revealed the unexpected presence of PKU in our patient, our multidisciplinary clinical team was instrumental in establishing our proband’s three different diagnoses.

## 4. Materials and Methods

Genetic testing was performed for diagnostic purposes after informed consent forms were obtained. The informed consent included authorization to publish significant results. CES was performed using the NGS Clinical Exome Solution^®^ v2 kit (SOPHiA Genetics, Saint-Sulpice, Switzerland) covering the coding regions and splice junctions of 4490 genes, using a paired-ends read mode with FastQ only analysis workflow on the NextSeq550DX^®^ NGS platform (Illumina, San Diego, CA, USA). The sequencing FastQ data were analyzed with the DDM^®^ platform by SOPHiA Genetics (https://www.sophiagenetics.com/technology/ accessed on 10 November 2023) to detect SNVs, indels, and CNVs. The patient’s mother DNA was also analyzed to assess inheritance. CMA analysis was performed using the GenetiSure CytoCGH Microarray kit (Agilent Technologies, Santa Clara, CA, USA), following the manufacturer’s instructions, using the ADM-2 algorithm for data analysis with Agilent CytoGenomics software (https://www.agilent.com/en/product/cgh-cgh-snp-microarray-platform/cgh-cgh-snp-microarray-software/cytogenomics-software-228500, accessed on 10 November 2023).

## Data Availability

All manuscript data are available.
